# Clinical and Electrophysiological Evaluation of Electrical Stimulation in Patients with Bell’s Palsy

**DOI:** 10.15388/Amed.2024.31.2.19

**Published:** 2024-12-04

**Authors:** Fatma Çelik, Aylin Ayyıldız, Selda Çiftci İnceoğlu, Figen Yılmaz, Mustafa Çelik, Banu Kuran

**Affiliations:** 1 The Balıklı Greek Hospital, Department of Physical Medicine and Rehabilitation, İstanbul, Turkey; 2 Ministry of Health, Başakşehir Çam and Sakura City Hospital, Department of Physical Medicine and Rehabilitation, İstanbul, Turkey; 3 University of Health Sciences, Şişli Hamidiye Etfal Training and Research Hospital, Department of Physical Medicine and Rehabilitation, İstanbul, Turkey; 4 Mustafa Çelik Clinic, Department of Otolaryngology, Istanbul, Turkey

**Keywords:** Bell’s palsy, depressive symptoms, electrical stimulation, electromyography, synkinesis, Raktažodžiai: Belo paralyžius, depresijos simptomai, elektrostimuliacija, elektromiografija, sinkinezė

## Abstract

**Background:**

The objective of this study is to assess the clinical and electrophysiological effectiveness of electrical stimulation in patients diagnosed Bell’s palsy (BP), and to examine its impact on potential complications that may arise during the course of the illness.

**Methods:**

Thirty patients diagnosed with BP were enrolled and randomized into two treatment groups of 15 patients each. The treatment group (TG) received 15 sessions of electrical stimulation (ES), infrared, and exercise therapy, 5 days a week for 3 weeks, while the control group (CG) received sham ES, infrared, and exercise therapy. Evaluations were conducted by electromyography before treatment and at 1 and 3 months after the end of treatment for all patients.

**Results:**

When evaluating patients in both groups based on the side where the BP developed, electrophysiological examination showed an increase in the rate of synkinesis in both the TG and CG. However, there was no statistically significant difference between the two groups. The study found that ES did not reduce the presence of synkinesis in denervated muscles. However, the nerve conduction studies of the studied branches of the facial nerve showed a decrease in distal latencies and an increase in amplitudes of compound muscle action potentials, indicating that ES has a positive effect on nerve regeneration.

**Conclusion:**

ES resulted in a notable reduction in distal latency values within the treatment group. Additionally, ES was found to significantly alleviate depressive symptoms, although it did not result in an improvement in overall quality of life. Therefore, ES is considered a safe treatment method that can be used in the treatment of BP.

## Abstract (it)

**Įvadas:**

šio tyrimo tikslas – įvertinti klinikinį ir elektrofiziologinį elektrostimuliacijos (ES) veiksmingumą pacientams, kuriems diagnozuotas Belo paralyžius (BP), ir ištirti jos poveikį galimoms komplikacijoms, kurių gali atsirasti ligos eigoje.

**Metodai:**

Trisdešimt pacientų, kuriems diagnozuotas BP, buvo įtraukti į dvi gydymo grupes po 15 pacientų. Gydomajai grupei (TG) buvo taikoma 15 elektrostimuliacijos (ES), infraraudonųjų spindulių ir mankštos terapija 5 dienas per savaitę tris savaites, o kontrolinei grupei (CG) – fiktyvi ES, infraraudonųjų spindulių ir mankštos terapija. Prieš gydymą ir praėjus vienam bei trims mėnesiams po gydymo pabaigos visi pacientai buvo vertinami elektromiografijos metodu.

**Rezultatai:**

Vertinant abiejų grupių pacientus pagal tai, kurioje pusėje išsivystė BP, elektrofiziologinis tyrimas parodė, kad tiek TG, tiek CG grupėje padidėjo sinkinezės dažnis. Tačiau statistiškai reikšmingo skirtumo tarp abiejų grupių nebuvo. Tyrimo metu nustatyta, kad ES nesumažino sinkinezės buvimo denervuotuose raumenyse. Tačiau tirtų veidinio nervo šakų nervinio laidumo tyrimai parodė, kad sumažėjo distalinės latencijos ir padidėjo sudėtinių raumenų veikimo potencialų amplitudės, o tai rodo, kad ES turi teigiamą poveikį nervų regeneracijai.

**Išvados:**

Dėl ES gydymo grupėje pastebimai sumažėjo distalinės latencijos vertės. Be to, buvo nustatyta, kad ES reikšmingai palengvino depresijos simptomus, nors ir nepagerino bendros gyvenimo kokybės. Todėl ES laikoma saugiu gydymo metodu, kurį galima taikyti gydant BP.

## Introduction

Bell’s palsy (BP) is a disorder characterized by the sudden onset of unilateral facial weakness. BP is the most common peripheral mononeuropathy of the cranial nerves. Although herpes simplex virus has been implicated as an etiologic factor in many studies, BP is essentially a diagnosis of exclusion and is considered a multifactorial disease. [1] New cases of BP are diagnosed at a rate of 11–53.3 per 100,000 people per year. [2] Studies report no significant difference in incidence between males and females in terms of gender distribution. [3] The incidence of BP peaks in the third and fourth decades, as well as in the sixth and seventh decades. The recurrence rate in cases with BP is reported to be between 2% and 7.3%. [4]

While the spontaneous recovery rate is reported to be 71–94% in BP cases that are not treated within the first 3 months, this rate increases to 82–95% in patients who receive any treatment. [5,6] The treatment of BP should be planned with a multidisciplinary approach to accelerate recovery and cure the disease without leaving any sequelae. Current treatments for BP include corticosteroids, antiviral medication, surgical intervention, electrical stimulation (ES), exercise, massage, botulinum toxin, and acupuncture. [7] The use of ES in the treatment of BP is a controversial topic. [8]

ES is a crucial treatment method that minimizes nerve paralysis atrophy and maintains the functionality of the contractile units of muscles. ES increases muscle metabolism and blood flow, similar to voluntary contractions. [8] Studies have shown that ES accelerates nerve regeneration and causes hypertrophy of muscle fibers. In the study conducted by Gutmann et al., histological examination of electrically stimulated muscles reported that ES delayed muscle atrophy. [9] Lal et al. conducted an experimental study where they applied ES to rats with traumatic facial nerve injury, resulting in accelerated recovery of the blink reflex. [10] Alakram and Puckree’s study reported that ES did not contribute to early BP recovery, but it could be used safely. [11]

The hypothesis of our study is to show that ES is an effective and beneficial treatment modality in patients with BP. The purpose of this study was to assess the impact of ES on the clinical characteristics of patients, as well as on BP sequelae and facial nerve electrophysiological parameters in patients diagnosed with BP.

## Methods

The study included 30 patients who were diagnosed with BP at the ENT clinic, and were referred to the Physical Medicine and Rehabilitation clinic. The study included patients aged 18 to 75 with facial paralysis grade 2–4 according to the House–Brackmann Scale (HBS). Patients with a history of recurrent facial paralysis on the same side, trauma, middle ear and parotid gland tumor surgery, history of cerebrovascular disease, diabetes mellitus, Lyme disease, varicella-zoster infection, intracranial mass, ear and parotid pathology, and temporal bone fracture were excluded. Each patient was receiving 3-week medical treatment which includes oral corticosteroid in appropriate dosage for BP and was referred to us within the first month after the onset of facial paralysis. This is a double-blind, randomized, controlled clinical trial conducted prospectively from April to July 2017. Ethical approval for the study was obtained from Hamidiye Etfal Training and Research Hospital (Decision year / number: 2017 / 772). The patients were informed about the content, purpose, and application of the study and their informed consent was obtained.

Patient randomization was performed by dividing them into two groups using a random number table. Both groups included 15 patients, and their demographic data were recorded. The TG received 15 sessions of ES, infrared, and exercise therapy, 5 days a week for 3 weeks. The CG received 15 sessions of sham ES, infrared, and exercise therapy, 5 days a week for 3 weeks. The study compared clinical findings and electrophysiological recordings of both groups of patients before and three months after treatment.

ES was applied to the motor points of 8 muscles innervated by the facial nerve (*m. frontalis, m. orbicularis oculi, m. corrugator, m. levator labii alaeque nasii, m. compressor naris, m. quadratus labii superioris and inferioris, m. orbicularis oris*). A total of 15 sessions were applied to each point, 3x30, 90 seconds, 5 days a week. As ES, intermittent galvanic current (COMPEX-3® Neuromuscular Electrical Stimulation Device, Belgium) with a pulse width of 100 milliseconds was applied. The 5x5 cm negative electrode was applied to the patient’s trapezius muscle on the same side as the lesion, and the 3 mm diameter positive pencil electrode was applied to the motor point of the muscles. In the sham ES application, the electrode was placed on the muscles but no electric current was applied. ES was performed by the same physician.

As exercise therapy, the patient was made to perform exercises in line with the movements of the muscles innervated by the facial nerve actively to the patient: raising eyebrows (*m. frontalis*), frowning (*m. corrugator*), closing eyes (*m. orbicularis oculi sup./inf*.), moving nose wings (*m. dilator naris*), smiling (*m. risorius*), showing teeth (*m. zygomaticus major/minor*), jaw tightening (*m. mentalis*), whistling (*m. orbicularis oris sup./inf*.) movements were given. If the patient could not move his muscles actively, these movements were performed passively. When active movements began, assisted active exercises were started, and after the muscle function was completed with active movements, resistant exercises were applied. All subjects were asked to repeat all exercises in front of a mirror in 10 sets, if possible 15 times a day. Exercise therapy was performed by the same physiotherapist.

All patients were evaluated electrophysiologically by another blind doctor with the NIHON KOHDEN MEB-9200K100124 EMG device (Tokyo, Japan). Motor nerve conduction studies and needle EMG recordings were taken. A standard concentric needle electrode (0.45 mm in diameter, 50 mm in length) was used for needle EMG recordings. The stimulation electrode was placed in front of the tragus and ES was applied. The ground electrode was placed on the forehead of the patient’s healthy side. In needle EMG recordings, electrical activities at rest and during voluntary activity were recorded while the needle electrode was in the *m. frontalis, m. orbicularis oculi* and *m. orbicularis oris* muscles. In the motor nerve conduction study in which the needle electrode was used as the recording electrode, distal latency and amplitude values were recorded. In needle EMG study, fibrillation potential and positive sharp wave (PSW) recordings during rest and motor unit potentials (MUP) fired during voluntary activity were recorded. The interference pattern was determined according to MUP intensity: 0, no MUP; 1, single oscillation; 2, forward-sparse; 3, sparse; 4, full interference. To evaluate the presence of synkinesis in the EMG study, while the needle electrode was in the patient’s orbicularis oculi muscle, active movement commands were given to the patient, such as laughing, showing teeth and pursing lips, and MUP recordings were taken. While the needle electrode was in the orbicularis oris muscle, voluntary active movement commands were given to the subject, such as eye closing and eyebrow raising, and MUP recordings were taken. Synkinesis rating based on MUP firing intensity was: 0, no MUP; 1, there is one MUP (mild synkinesis); 2, there are two to four MUPs (moderate synkinesis); 3, there were more than four MUPs (severe synkinesis).

The mood of the patients was assessed using the Beck Depression Inventory (BDI), while the quality of life was assessed using the Short Form-36 (SF-36). The affected side of all patients was scored according to the HBS system for the degree of paralysis, presence of crocodile tears, contracture, and hemifacial spasm.

Statistical analysis was performed using NCSS® (Number Cruncher Statistical System) 2007 (Kaysville, Utah, USA). Descriptive statistical methods, including mean, standard deviation, median, frequency, ratio, minimum, and maximum, were used to evaluate the study data. The distribution of variables was measured using the Kolmogorov–Smirnov test. Independent quantitative data was analyzed using the Mann–Whitney U test, while quantitative dependent data was analyzed using the Wilcoxon test. Qualitative independent data was analyzed using the Chi-square test, and the Fisher test was used when the conditions for the Chi-square test were not met. Significance was assessed at the p<0.05 level.

## Results

The study included 30 patients, with 15 patients in each group. The mean age of all patients was 45.25±16.53 (23–69) years. The TG group comprised of 11 male and 4 female patients, with a mean age of 43.86±14.82 (24–69) years. The CG group comprised of 7 male and 8 female patients, with a mean age of 46.60±18.01 (23–67) years (Table 1).

**Table 1:** Demographic Characteristics of the Groups

		Treatment Group (n=15)			Control Group (n=15)			p	
		Mean±SD /n-%			Mean±SD /n-%				
**Age**		43.9	±	14.8	46.6	±	18.0	0.740	m
**Sex**	Male	11		73.33%	7		46.7%	0.136	X^2^
	Female	4		26.7%	8		53.3%		
**Affected Side**	Right	5		33.3%	3		20.0%	0.409	X^2^
	Left	10		66.7%	12		80.0%		

m Mann–Whitney u test / X^2^ Chi-square test (Fischer test)

In the TG group, 5 patients (33.3%) had BP on the right side, while 10 patients (66.7%) had BP on the left side. In the CG group, 3 patients (20.0%) had BP on the right side, and 12 patients (80.0%) had BP on the left side. There was no statistically significant difference (p>0.05) between the groups regarding the affected side. There was no statistically significant difference in mean age, gender, or comorbidities between the groups (p>0.05) (Table 1). No adverse effects were observed during the study.

There was no statistically significant difference found between TG and CG in the development of crocodile tears and hemifacial spasm, or in the distribution of HBS grades (Table 2) before treatment, 1 month, and 3 months (p>0.05). Additionally, none of the patients developed a contracture.

**Table 2:** Comparison of groups according to the House–Brackmann Scale

		Treatment Group		Control Group		p	
		n	%	n	%		
**House–Brackmann Scale**							
**Pretreatment**	I	0	0.0%	0	0.0%	0.660	X^2^
	II	5	33.3%	9	60.0%		
	III	3	20.0%	4	26.7%		
	IV	7	46.7%	2	13.3%		
**3rd Month**	0	1	6.7%	0	0.0%	0.142	X^2^
	I	7	46.7%	6	40.0%		
	II	3	20.0%	8	53.3%		
	III	4	26.7%	1	6.7%		

X^2^ Chi-square test (Fischer test)

No statistically significant difference was found in the distal latency value of the temporal branch of the n. facialis between the pretreatment and 3-month posttreatment groups (p>0.05) in the electrophysiological evaluations. Although the distal latency values of the temporal branch of the n. facialis showed a statistically significant change in TG compared to the pretreatment period, there was no statistically significant change in CG (p=0.001, p=0.054, respectively). However, this change in distal latency value seen in TG was not statistically significant compared to CG (p>0.05). There was no statistically significant difference found between the TG and CG in the changes of the compound muscle action potential (CMAP) amplitude (p>0.05) (Table 3).

**Table 3:** Comparison of distal latency and CMAP amplitude values obtained from the temporal, zygomatic and buccal branches of n. facialis

	Treatment Group (n=15)		Control Group (n=15)		p
	Mean±SD	Median	Mean±SD	Median	
***Distal latency of temporal branch of n. facialis (Ms)***					
Pretreatment	5.4 ± 1.2	4.9	5.2± 1.0	5.3	0.965 ^m^
3rd Month	4.8 ± 1.1	4.5	4.7 ± 0.6	4.8	0.913 ^m^
3rd Month Change	0.5 ± 0.4	0.5	0.4 ± 0.8	0.4	0.777 ^m^
*Intragroup Change p*	**0.001** ^w^		0.054 ^w^		
***CMAP Amplitude of temporal branch of n. facialis (Mv)***					
Pretreatment	1.4 ± 1.0	1.0	1.6 ± 1.2	1.3	0.917 ^m^
3rd Month	1.7 ± 1.0	1.7	2.4± 1.0	2.5	0.081 ^m^
3rd Month Change	0.2 ± 0.7	0.4	0.8 ± 1.6	0.5	0.254 ^m^
*Intragroup Change p*	0.191 ^w^		0.064 ^w^		
***Distal Latency of the zygomatic branch of n. facialis (Ms)***					
Pretreatment	4.7 ± 1.6	4.2	3.7 ± 1.0	3.5	0.052 ^m^
3rd Month	3.4 ± 0.8	3.3	3.3 ± 0.7	3.3	0.612 ^m^
3rd Month Change	1.3 ± 1.3	1.1	0.4 ± 0.9	0.3	0.029 ^m^
*Intragroup Change p*	0.001 ^w^		0.094 ^w^		
***CMAP Amplitude of the zygomatic branch of n. facialis***					
Pretreatment	0.7 ± 0.6	0.5	1.6 ± 1.0	1.5	0.052 ^m^
3rd Month	1.5 ± 1.2	1.1	2.6 ± 1.1	2.6	0.065 ^m^
3rd Month Change	0.9 ± 0.9	0.8	1.0 ± 1.3	0.9	0.74 ^m^
*Intragroup Change p*	**0.002** ^w^		**0.006** ^w^		
***Distal Latency of the buccal branch of n. facialis (Ms)***					
Pretreatment	6.1 ± 2.1	5.5	5.4 ± 2.1	5.3	0.643 ^m^
3rd Month	4.5 ± 1.2	4.2	4.9 ± 1.6	5.0	0.084 ^m^
3rd Month Change	1.1 ± 2.3	1.0	0.5 ± 1.7	0.0	0.174 ^m^
*Intragroup Change p*	**0.002** ^w^		0.289 ^w^		
***CMAP Amplitude of the buccal branch of n. facialis***					
Pretreatment	0.8 ± 1.1	0.4	0.8 ± 0.9	0.5	0.533 ^m^
3rd Month	1.9 ± 1.6	1.3	1.7 ± 1.7	1.1	0.547 ^m^
3rd Month Change	1.1 ± 1.1	0.9	0.8 ± 1.8	0.5	0.351 ^m^
*Intragroup Change p*	**0.002** ^w^		0.026 ^w^		

m Mann–Whitney U Test , ^w^ Wilcoxon Test, SD: Standard deviation, CMAP: Compound Muscle Action Potential

There were no statistically significant differences between the TG and CG groups in terms of distal latency values and CMAP amplitude values of the zygomatic branch of the n. facialis recorded from the m. orbicularis oculi muscle before and after treatment at month 3 (p>0.05). Additionally, in the TG group, there was no statistically significant difference in the distal latency values of the zygomatic branch of the n. facialis recorded from the m. orbicularis oculi muscle before and after treatment at month 3 (p>0.05). Although the distal latency values of the branch decreased significantly (p=0.001) after treatment, no statistically significant change was observed in the CG (p=0.094). In the TG, the distal latency values of the zygomatic branch of the facial nerve were significantly higher than those in the CG at month 3 after treatment (p=0.029). In both the TG and CG, there was a statistically significant increase in the amplitude values of the zygomatic branch of the facial nerve (n. facialis) at month 3 compared to pretreatment (p<0.05). However, there was no statistically significant difference in the change of amplitude values of the orbicularis oculi muscle (m. orbicularis oculi) at month 3 between TG and CG (p>0.05) (Table 3).

There was no statistically significant difference found between the pretreatment and 3-month posttreatment groups in the distal latency and CMAP amplitude values of the buccal branch of the n. facialis recorded from the m. orbicularis oris muscle (p>0.05). However, the distal latency values of the buccal branch of n. facialis in the 3rd month of TG showed a significant decrease compared to the pre-treatment, while it did not show a statistically significant change in CG (p=0.002, p=0.289, respectively). In both groups, the amplitude values of the buccal branch of the n. facialis increased significantly compared to the pretreatment period at month 3 (p<0.05). However, there was no statistically significant difference in the change of distal latency and CMAP amplitude values of the buccal branch of n. facialis between the TG and CG at month 3 compared to before treatment (p>0.05) (Table 3).

In both groups, there were no significant differences in fibrillation potential and PSW potentials, as well as interference distribution in the m. frontalis, m. orbicularis oculi, and m. orbicularis oris muscles before and after the 3rd month of treatment (p>0.05) (Table 4).

**Table 4:** Comparison of Fibrillation Potential and Positive sharp wave potentials of *M. Frontalis, M. Orbicularis Oculi* and *M. Orbicularis Oris* muscles of the groups

		Treatment Group (n=15)		Control Group (n=15)		p	
		n	%	n	%		
*Fibrillation of M. Frontalis*							
Pretreatment	0	4	26.7%	5	33.3	0.690	
	+1	6	40.0%	6	40.0%		
	+2	5	33.3%	3	20.0%		
	+3	0	0.0%	1	6.7%		
3rd Month	0	13	86.7%	12	80.0%	0.624	X^2^
	+1	2	13.3%	3	20.0%		
*PSW of M. Frontalis*							
Pretreatment	0	4	26.7%	6	40.0%	0.439	X^2^
	+1	6	40.0%	6	40.0%		
	+2	5	33.3%	2	13.3%		
	+3	0	0.0%	1	6.7%		
3rd Month	0	12	80.0%	12	80.0%	1.000	X^2^
	+1	3	20.0%	3	20.0%		
*Fibrillation of M. Orbicularis Oculi*							
Pretreatment	0	3	20.0%	5	33.3%	0.409	X^2^
	+1	8	53.3%	8	53.3%		
	+2	4	26.7%	2	13.3%		
3rd Month	0	10	66.7%	14	93.3%	0.068	X^2^
	+1	4	26.7%	1	6.7%		
	+2	1	6.7%	0	0.0%		
*PSW of M. Orbicularis Oculi*							
Pretreatment	0	5	33.3%	4	26.7%	0.690	X^2^
	+1	6	40.0%	9	60.0%		
	+2	4	26.7%	2	13.3%		
3rd Month	0	10	66.7%	14	93.3%	0.068	X^2^
	+1	5	33.3%	1	6.7%		
*Fibrillation of M. Orbicularis Oris*							
Pretreatment	0	1	6.7%	3	20.0%	0.409	X^2^
	+1	8	53.3%	4	26.7%		
	+2	6	40.0%	7	46.7%		
	+3	0	0.0%	1	6.7%		
3rd Month	0	10	66.7%	12	80.0%	0.068	X^2^
	+1	4	26.7%	3	20.0%		
	+2	1	6.7%	0	0.0%		
*PSW of M. Orbicularis Oris*							
Pretreatment	0	1	6.7%	3	20.0%	0.690	X^2^
	+1	8	53.3%	5	33.3%		
	+2	6	40.0%	6	40.0%		
	+3	0	0.0%	1	6.7%		
3rd Month	0	9	60.0%	12	80.0%	0.068	X^2^
	+1	4	26.7%	3	20.0%		
	+2	2	13.3%	0	0.0%		

X^2^ Chi-square test (Fischer test)

PSW: Positive Sharp Wave

Before and 3 months after treatment, there were no statistically significant differences in mouth synkinesis with eye closing, mouth synkinesis with eyebrow raising, eye synkinesis with smiling, eye synkinesis with teeth showing, and eye synkinesis with lip pursing between the TG and CG (p>0.05) (Table 5).

**Table 5:** Comparison of the synkinesis distribution of groups caused by certain movements

		Treatment Group (n=15)		Control Group (n=15)		p
		n	%	n	%	
***Oral Synkinesis with Eye Closing***						
Pretreatment	No MUP	6	40	5	33.3	0.705 X^2^
	Mild	1	6.70	2	13.3	
	Moderate	6	40	5	33.3	
	Severe	2	13.3	3	20	
3rd Month	No MUP	3	20	4	26.7	0.666 X^2^
	Mild	0	0	1	6.7	
	Moderate	6	40	4	26.7	
	Severe	6	40	6	40	
***Oral Synkinesis with Eyebrow Lifting***						
Pretreatment	No MUP	13	86.7	12	80	0.524 X^2^
	Mild	0	0	2	13.3	
	Moderate	1	6.7	1	6.7	
	Severe	1	6.7	0	0	
3rd Month	No MUP	8	53.3	9	60	0.713 X^2^
	Mild	1	6.7	1	6.7	
	Moderate	5	33.3	4	26.7	
	Severe	1	6.7	1	6.7	
***Eye Synkinesis with Laughing***						
Pretreatment	No MUP	5	33.3	2	13.3	0.195 X^2^
	Mild	0	0	0	0	
	Moderate	5	33.3	5	33.3	
	Severe	5	33.3	8	53.3	
3rd Month	No MUP	1	6.7	0	0	0.309 X^2^
	Mild	1	6.7	0	0	
	Moderate	7	46.7	4	26.7	
	Severe	6	40	11	73.3	
***Eye Synkinesis with Teeth Showing***						
Pretreatment	No MUP	5	33.3	1	6.7	0.068 X^2^
	Mild	1	6.7	1	6.7	
	Moderate	1	6.7	4	26.7	
	Severe	8	53.3	9	60	
3rd Month	Moderate	7	46.7	7	46.7	1.00 X^2^
	Severe	8	53.3	8	53.3	
***Eye Synkinesis with Pursing Lips***						
Pretreatment	No MUP	10	66.7	8	53.3	0.456 X^2^
	Mild	1	6.7	2	13.3	
	Moderate	3	20	3	20	
	Severe	1	6.7	2	13.3	
3rd Month	No MUP	7	46.7	5	33.3	0.456 X^2^
	Mild	2	13.3	1	6.7	
	Moderate	2	13.3	5	33.3	
	Severe	4	26.7	4	26.7	

X^2^ Chi-square test (Fischer test)

There was no statistically significant difference in BDI between the TG and CG before treatment (p>0.05). When the evaluations before and after treatment were compared at the 3rd month, there was a significant difference in both groups (p<0.05). This significant difference was significantly greater in the treatment group (p<0.05) (Table 6).

**Table 6:** Comparison of groups in terms of Beck Depression Inventory Scores

	Treatment Group (n=15)				Control Group (n=15)				p	
	Mean ± SD			Median	Mean ±S D			Median		
***Beck Depression Inventory***										
Pretreatment	12.7	±	9.9	10.0	11.4	±	6.3	11.0	0.917	^m^
3rd Month	3.3	±	2.3	4.0	8.2	±	5.3	9.0	***0.008***	^m^
3rd Month Change	9.4	±	8.0	8.0	3.2	±	3.5	4.0	***0.015***	^m^
*Intra-group Change p*	***0.001***			^w^	***0.007***			^w^		

m Mann-Whitney u test / w Wilcoxon test

There was no statistically significant difference in the SF-36 role limitations due to emotional problems, energy/fatigue, emotional well-being, and social functioning scores between TG and CG before treatment and 3 months (p>0.05). Additionally, both groups showed a statistically significant increase in the SF-36 role limitations due to emotional problems, emotional well-being, and social functioning scores at the 3rd month compared to pretreatment (p<0.05). When these significant changes were compared between the two groups, no significant difference was found (p>0.05) (Table 7).

**Table 7:** Comparison of groups in terms of Short Form-36 Sub-parameter

	Treatment Group (n=15)		Control Group (n=15)		p
	Mean ± SD	Median	Mean ± SD	Median	
***Role limitations due to emotional problems***					
Pretreatment	30.9 ± 31.9	33.0	29.8 ± 30.9	33.0	0.948 ^m^
3rd Month	88.8 ± 27.3	100.0	79.7 ± 24.8	100.0	0.155 ^m^
3rd Month Change	57.9 ± 31.9	67.0	49.9 ± 33.9	66.0	0.374 ^m^
*Intra-group Change p*	**0.001** ^w^		**0.002** ^w^		
***Energy/fatigue***					
Pretreatment	50.0 ± 21.4	50.0	44.7 ± 16.5	40.0	0.558 ^m^
3rd Month	69.3 ± 17.4	70.0	58.7 ± 18.7	55.0	0.162 ^m^
3rd Month Change	19.3 ± 15.0	20.0	14.0 ± 14.9	15.0	0.477 ^m^
*Intra-group Change p*	**0.001** ^w^		**0.012** ^w^		
***Emotional well-being***					
Pretreatment	60.8 ± 21.8	52.0	62.1 ± 15.0	64.0	0.787 ^m^
3rd Month	81.6 ± 10.1	80.0	74.9 ± 12.2	76.0	0.133 ^m^
3rd Month Change	20.8 ± 16.0	28.0	12.8 ± 7.6	12.0	0.117 ^m^
*Intra-group Change p*	**0.002** ^w^		**0.001** ^w^		
***Social functioning***					
Pretreatment	43.1 ± 23.5	37.0	44.7 ± 20.4	50.0	0.642 ^m^
3rd Month	87.3 ± 15.8	100.0	78.1 ± 13.0	75.0	0.082 ^m^
3rd Month Change	44.2 ± 27.1	50.0	33.3 ± 16.8	25.0	0.198 ^m^
*Intra-group Change p*	**0.001** ^w^		**0.001** ^w^		

m Mann–Whitney u test, ^w^ Wilcoxon test

## Discussion

Studies investigating the effect of ES on BP prognosis have reported mixed results in the literature. Balliet argued that ES may be contraindicated, stating that it only helps the patient’s motivation by acting as a placebo during nerve recovery. Additionally, ES applied to the denervated muscle increases the development of fibril degeneration, synkinesis, and fibrosis, causing a delay in reinnervation. [12] Cederwall et al. argued that ES may have a destructive effect on reinnervation in cases of BP and should be considered a contraindication for facial nerve disease. [13] Teixeira et al. reported in their review article that ES did not provide a significant benefit over placebo in BP. [14] However, Targan et al. reported significant improvements in the degree of HBS and motor conduction latency abnormalities in chronic BP cases when administered low-intensity, monophasic, short-duration electrical current. Particularly after regular ES treatment, a significant decrease in motor conduction latency was observed, emphasizing that this was an objective indicator of nerve innervation. The authors noted that there were no significant improvements in clinical findings such as synkinesis, lacrimation, and drooling. [15] In our study, especially distal latency was evaluated and although a significant difference was shown only in the zygomatic branch compared to the control group, there was a significant difference in the treatment group in the evaluations within the group before and after treatment. In the study conducted by Tuncay et al., a significant increase in CMAP amplitude and a significant decrease in latency were found in patients with BP who received ES treatment compared to the control group, [8] and these data are consistent with our study. Similarly, Hyvarinen et al. applied ES to patients diagnosed with BP and compared them with the healthy side in their study and found a significant improvement in the latency of the upper branch of the facial nerve on the affected side after treatment. [16] In their experimental animal study, Foecking et al. reported that daily ES increased facial nerve regeneration and facilitated and enhanced improvement in facial nerve function. [17]

In EMG, amplitude is specifically related to the number of muscle fibers innervated. It has been shown that especially in BP patients, the amplitude is higher on the paralyzed side compared to the healthy side. [18] Similarly, a long latency of the action potential is an indicator of pathology, and shortening of the latency is associated with regeneration and electrophysiological recovery. In the animal study conducted by Deng et al., it was shown that ES was effective in nerve regeneration with an increase in amplitude and a decrease in latency in rats diagnosed with BP. [19] Preston et al. defined normal distal latency and amplitude of the facial nerve in their textbook. These were defined as amplitudes greater than and equal to 1.0 mV in the buccal and zygomatic branches of the facial nerve, and distal latency as less than and equal to 3.1 ms for the zygomatic branch. [20] In our study, we observed that in the evaluation of the amplitude of the zygomatic and buccal branches, the pretreatment pathologically observed values in the ES treatment group normalized as posttreatment. In the evaluation of distal latency of the zygomatic branch, although we did not see that the pretreatment pathological values reached normal values after treatment, we observed that they approached normal.

Synkinesis is the most common sequela of BP. Different rates of synkinesia development after BP have been reported in the literature. Taverner reported a frequency of 55%, while Yamamoto et al. reported a frequency of 18.3%. [21,22] Currently, there are no studies in the literature that demonstrate the superiority of any treatment modality in reducing synkinesis. Nakamura et al. reported that the biofeedback method is effective in reducing the rate of synkinesis that develops after facial paralysis. [23] In their study, Cronin et al. reported that the combination of neuromuscular facial training and EMG was effective in rehabilitating facial paralysis and reducing synkinesia. [24] There are limited studies investigating the effect of ES on synkinesis. Yaltırık et al. reported a lower rate of synkinesis development, contractures, and other sequelae in cases where ES was used compared to cases where ES was not used. [25] Synkinesis was assessed electrophysiologically in all cases three months after treatment. Our study’s results showed an increase in the rate of synkinesis in both the TG and CG, but no statistically significant difference was found between them. Our study found that ES did not reduce synkinesis in denervated muscles. Interestingly, both groups showed an increase in synkinesis, which may indicate that synkinesis tends to increase during the healing process of facial nerve damage, particularly in the third month. Furthermore, it was noted that patients in the TG did not experience a higher incidence of complications, such as crocodile tears, contractures, or hemifacial spasms.

According to a study published in 2020, BP can lead to increased levels of anxiety and depression symptoms, which can negatively affect the patient’s quality of life. The authors think that this is particularly related to the change in the patient’s appearance. [26] Quality of life scores have been found to be associated with HBS and other facial palsy scores in long-term follow-up. [27] The decrease in BDI scores was found to be significantly greater in the TG than in the CG, as evidenced by the results of our study. It is postulated that ES has the additional effect of reducing depressive symptoms and enhancing patients’ confidence in the efficacy of the treatment. Nevertheless, no impact of ES treatment on quality of life was discerned in patients with a BP diagnosis. This is a situation that is particularly contradictory. For these results, which were obtained with 2 scales evaluating emotional states, there is a need for studies with larger patient groups in the future. A review of the literature revealed no other studies that had evaluated the impact of ES treatment on the quality of life and depressive symptoms in patients with BD. It is our contention that this study will make a significant contribution to the existing literature on the subject.

The study has limitations due to the relatively small number of cases included, and also the evaluation of mood changes using BDI scores, so the inability to make further interpretations for the diagnosis of depression.

## Conclusion

The findings indicated that while ES resulted in a notable reduction in distal latency values within the treatment group, but there was no statistically significant improvement when compared to the control group. Additionally, ES was found to significantly alleviate depressive symptoms, although it did not result in an improvement in overall quality of life. Therefore, ES is considered a safe treatment method that can be used in the treatment of BP.

## Data Availability

The data that support the findings of this study are available from the corresponding author, [author initials], upon reasonable request.

## References

[1] Hohman MH, Hadlock TA (2014). Etiology, diagnosis, and management of facial palsy: 2000 patients at a facial nerve center. Laryngoscope.

[2] De Diego JI, Prim MP, Madero R, Gavilán J (1999). Seasonal patterns of idiopathic facial paralysis: a 16-year study. Otolaryngol Head Neck Surg.

[3] Katusic SK, Beard CM, Wiederholt WC, Bergstralh EJ, Kurland LT, Incidence clinical features and prognosis in Bell’s palsy Rochester Minnesota 1968-1982 (1986). Ann Neurol.

[4] Pereira LM, Obara K, Dias JM, Menacho MO, Lavado EL, Cardoso JR (2011). Facial exercise therapy for facial palsy: systematic review and meta-analysis. Clin Rehabil.

[5] Ferreira M, Marques EE, Duarte JA, Santos PC (2015). Physical therapy with drug treatment in Bell palsy: a focused review. Am J Phys Med Rehabil.

[6] Eviston TJ, Croxson GR, Kennedy PG, Hadlock T, Krishnan AV (2015). Bell’s palsy: aetiology, clinical features and multidisciplinary care. J Neurol Neurosurg Psychiatry.

[7] Nicastri M, Mancini P, De Seta D (2013). Efficacy of early physical therapy in severe Bell’s palsy: a randomized controlled trial. Neurorehabil Neural Repair.

[8] Tuncay F, Borman P, Taşer B, Ünlü İ Samim E (2015). Role of electrical stimulation added to conventional therapy in patients with idiopathic facial (Bell) palsy. Am J Phys Med Rehabil.

[9] Gutmann E, Guttmann L (1944). The Effect of Galvanic Exercise on Denervated And Re-Innervated Muscles in the Rabbit. J Neurol Psychiatry.

[10] Lal D, Hetzler LT, Sharma N (2008). Electrical stimulation facilitates rat facial nerve recovery from a crush injury. Otolaryngol Head Neck Surg.

[11] Alakram P, Puckree T (2010). Effects of electrical stimulation on House-Brackmann scores in early Bell’s palsy. Physiother Theory Pract.

[12] Balliet R, Payton OD (1989). Facial paralysis and other neuromuscular dysfunctions of the peripheral nervous system. *Manual of Physical Therapy*.

[13] Cederwall E, Olsén MF, Hanner P, Fogdestam I (2006). Evaluation of a physiotherapeutic treatment intervention in “Bell’s” facial palsy. Physiother Theory Pract.

[14] Teixeira LJ, Valbuza JS, Prado GF (2011). Physical therapy for Bell’s palsy (idiopathic facial paralysis). Cochrane Database Syst Rev.

[15] Targan RS, Alon G, Kay SL (2000). Effect of long-term electrical stimulation on motor recovery and improvement of clinical residuals in patients with unresolved facial nerve palsy. Otolaryngol Head Neck Surg.

[16] Hyvärinen A, Tarkka IM, Mervaala E, Pääkkönen A, Valtonen H, Nuutinen J (2008). Cutaneous electrical stimulation treatment in unresolved facial nerve paralysis: an exploratory study. Am J Phys Med Rehabil.

[17] Foecking EM, Fargo KN, Coughlin LM, Kim JT, Marzo SJ, Jones KJ (2012). Single session of brief electrical stimulation immediately following crush injury enhances functional recovery of rat facial nerve. J Rehabil Res Dev.

[18] Guntinas-Lichius O, Volk GF, Olsen KD (2020). Facial nerve electrodiagnostics for patients with facial palsy: a clinical practice guideline. Eur Arch Otorhinolaryngol.

[19] Deng Y, Xu Y, Liu H (2018). Electrical stimulation promotes regeneration and re-myelination of axons of injured facial nerve in rats. Neurol Res.

[20] Preston DC, Shapiro BE (2012). Electromyography and Neuromuscular Disorders E-Book: Clinical-Electrophysiologic Correlations (Expert Consult - Online).

[21] Taverner D (1955). Bell’s palsy: a clinical and electromyographic study. Brain.

[22] Yamamoto E, Nishimura H, Hirono Y (1988). Occurrence of sequelae in Bell’s palsy. Acta Otolaryngol Suppl.

[23] Nakamura K, Toda N, Sakamaki K, Kashima K, Takeda N (2003). Biofeedback rehabilitation for prevention of synkinesis after facial palsy. Otolaryngol Head Neck Surg.

[24] Cronin GW, Steenerson RL (2003). The effectiveness of neuromuscular facial retraining combined with electromyography in facial paralysis rehabilitation. Otolaryngol Head Neck Surg.

[25] Yaltirik H, On A, Kirazli Y (2001). Perİferİk Fasİyal Paralİzİde Elektİrİk Stİmulasyonunun Fonksİyonel İyİleŞme Ve Komplİkasyon GelİŞİmİ Üzerİne Etkİlerİ [Effects of Electrical Stimulation on Functional Recovery and Occurrence of Complications in Peripheric Facial Paralysis]. Ege Fiz Tip Reh Der.

[26] Cuenca-Martínez F, Zapardiel-Sánchez E, Carrasco-González E, La Touche R, Suso-Martí L (2020). Assessing anxiety, depression and quality of life in patients with peripheral facial palsy: a systematic review. PeerJ.

[27] Bylund N, Hultcrantz M, Jonsson L, Marsk E (2021). Quality of Life in Bell’s Palsy: Correlation with Sunnybrook and House-Brackmann Over Time. Laryngoscope.

